# Identification of allergic rhinitis-related genes and mediating immune cells based on cis-eQTL: A Mendelian randomization study

**DOI:** 10.1097/MD.0000000000048349

**Published:** 2026-04-17

**Authors:** Jianghua Peng, Xin Yan, Mingzhu Shen, Junmei Xuan, Suhua Chen

**Affiliations:** a Department of General Practice, Shaoxing People’s Hospital (The First Affiliated Hospital, Shaoxing University), Shaoxing, China; b Department of Otolaryngology, Shaoxing People’s Hospital (The First Affiliated Hospital, Shaoxing University), Shaoxing, China; c Department of Pharmacy, Shaoxing People’s Hospital (The First Affiliated Hospital, Shaoxing University), Shaoxing, China.

**Keywords:** allergic rhinitis, eQTL, gene, immune cells, Mendelian randomization

## Abstract

In this study, we aimed to identify genes causally associated with allergic rhinitis (AR) by extracting cis-expression quantitative trait loci (cis-eQTL) as instrumental variables using the Mendelian randomization (MR) method, and further explore the role mechanisms of gene-related immune cells in AR. All genome-wide association study (GWAS) and eQTL data were from public databases. Genes causally associated with AR were screened by the summary-data-based Mendelian randomization (SMR) method to obtain Gene set 1. Predicted AR-related genes from 5 databases were combined and intersected with Gene set 1 to get Gene set 2. Genes in Gene set 2 with H4 posterior probability > .75 were selected through colocalization analysis to obtain Gene set 3. Then, 2-step 2-sample MR analysis was used to identify potential mediating immune cells. Gene set 1 had 29 genes. Gene set 2 contained 10 genes after intersection. Gene set 3 included TNFRSF18, HCP5, and CD44. MR mediation analysis showed TNFRSF18 could be a significant exposure, and CD3 on CD39+ activated Treg and CD4+ Treg could be mediators. TNFRSF18 negatively regulated AR and positively regulated the 2 immune cells, while immune cells negatively regulated AR. TNFRSF18, HCP5, and CD44 are significantly causally associated with the development of AR and may serve as drug targets. Among them, TNFRSF18 is more strongly supported. It may inhibit AR progression by upregulating CD3 on CD39+ activated Treg and CD4+ Treg, highlighting the role of these immune cells in the gene-mediated protection against AR.

## 1. Introduction

Allergic rhinitis (AR) is an inflammatory response of the nasal mucosa in susceptible individuals after exposure to allergens, mediated mainly by immunoglobulin E (IgE), with the involvement of a variety of immunoreactive cells and cytokines in the body.^[1]^ Globally, about 10% to 40% of patients suffer from AR, and in recent years the incidence of AR has been increasing worldwide due to changes in living as well as ecological conditions caused by air pollution.^[2]^ Allergic rhinitis can develop at any age, and the disease is estimated to affect 10% to 30% of adults and 40% of children, and its incidence has been increasing annually worldwide.^[3]^ Although AR is not fatal, its characteristic clinical features and complications, such as sinusitis and asthma, can affect people’s quality of life and social functioning, resulting in significant physical health and psychological burden.^[4]^ Meanwhile, AR also has significant health and economic impact through its use of resources for education, productivity, and health care.^[5]^

Although desensitization therapy and surgical treatment have significant therapeutic effects on some patients with AR, AR is still primarily treated with medication. Commonly used medications include oral antiallergic drugs and nasal steroids. However, about one-third of patients still have insignificant effects after medical treatment.^[6]^ Therefore, the search for new drug targets and treatment methods are of great importance. Reports suggest that genetic and environmental factors may play important roles in the progression of AR.^[7]^ We plan to use Mendelian randomization (MR) analysis to search for effective treatments for AR at the genetic level.

Compared with observational studies, Mendelian randomization (MR) study can maximize the removal of confounding factors and use single nucleotide variations (SNPs) as instrumental variables to search for causal relationships between exposure and outcome at the genetic level.^[8]^ Summary data based Mendelian randomization (SMR) combines genome-wide association study (GWAS) data with expression quantitative trait locus (eQTL) and was also used in our work to search for causal variations mediated by gene expression.^[9]^ Colocalization evaluates whether 2 traits are influenced by the same or different causal variations, and we were interested in exposure and outcome at a shared causal variant.^[10]^ This article aimed to identify genes related to the pathogenesis of AR by MR analysis of cis-eQTL. The parallel colocalization method is used to obtain new drug targets for the treatment of AR. By MR analysis of immune cells, the mechanism of gene action on AR by immune cells will be further investigated.

## 2. Materials and methods

### 2.1. Data collection

The GWAS data of AR were obtained from the FinnGen R10 database and used as the outcome variable (number of cases: 12,240; number of controls: 3,92,069). The cis-expression quantitative trait loci (cis-eQTL) data in blood were downloaded from the eQTLGen database to serve as the exposure variable. We queried genes predicted to be associated with AR from 5 databases, including the Therapeutic Target Database (TTD), the Pharmacogenomics Knowledgebase (PharmGkb), GeneCards, DrugBank, and the DisGeNET database. Among them, genes retrieved from GeneCards had a relevance score >1. The genome-wide association data for 731 immune cell traits were sourced from the study by Orrù’s team.^[11]^ All the data were from European populations. The URLs of the abovementioned databases are listed in Table S1, Supplemental Digital Content, https://links.lww.com/MD/R707.

### 2.2. Gene set 1

We performed SMR analysis of cis-eQTLs from eQTLGen database with AR data downloaded from FinnGen R10 database, along with the HEIDI test, and corrected the *P*-values of SMR results with FDR. Genes with *P*-values >.05 in the HEIDI test and *P*-values <.05 in the FDR correction were obtained as having a significant causal relationship with AR in Gene set 1.

### 2.3. Gene set 2

The genes related to AR predicted by the 5 databases were included in the concatenation set, and then the concatenation set was intersected with Gene set 1 to obtain Gene set 2.

### 2.4. Gene set 3

Based on 100 kb fragments of the upstream and downstream genes, colocalization analysis of genes and AR was performed in Gene set 2. The posterior probability of H4 (the hypotheses of association with both traits at a shared causal variant) > 0.75 is the threshold for the shared genetic effect between the 2 traits, then a colocalization relationship was considered to exist and positive genes were obtained in Gene set 3.

### 2.5. MR for mediation of immune cells

A 2-step, 2-sample MR for the mediation analysis was used to screen for genes related to AR and immune cells that could serve as mediators. *Step 1*: The genes in Gene set 3 were used as exposures, and SNPs of 1000 kb fragments upstream and downstream of the genes were extracted as instrumental variables to screen for genes associated with AR by a bidirectional MR analysis; *Step 2*: We used the 731 traits of immune cells as exposures and AR as the outcome to screen for immune cells associated with AR; *Step 3*: Using the genes obtained in step 1 as exposures and the traits of immune cells obtained in step 2 as outcomes, a 2-sample MR analysis was performed again. If for the same gene and the same trait of immune cell, the SNPs used as instrumental variables in step 1 were duplicated with those in step 2, the duplicate SNPs were deleted in step 2, and step 2 was performed again. Five methods were used in each step of the MR analysis: Egger, weighted median, inverse variance weighted (IVW), simple mode, and weighted mode. Assuming that the *P*-value threshold of MR analysis by IVW prevailed, and the results of combining the 5 MR analysis methods acted in the same direction, that is, the odds ratios (ORs) were all greater than or <1, a causal relationship between the exposure and the outcome was considered to exist. About 95% confidence interval (CI) was also used to describe the results. And the MR Egger method was used to exclude the pleiotropy of the results. *Step 4*: The effect value of genes on AR with MR analysis in step 1 was named beta_c, the effect value of immune cells to AR was named beta_b, the effect value of genes to immune cells was named beta_a, the direct effect of genes to AR was named beta_dir, the mediating effect was named beta_ab, and the percentage of mediating effect was named beta_p. “Leave-one-out” sensitivity analysis was used to identify the presence of outlier SNPs. The clumping conditions used for each step are shown in Table 1.

**Table 1:** The filtering criteria at each step of MR analysis for mediation.

Steps of MR for mediation	Filter criteria for clumping SNPs as instrumental variables	Filter criteria for *P*-value of IVW method
Gene to AR	*P*^1^ < 5e−6, kb = 10,000, *r*^2^ < 0.1, maf > 0.01, *F* statistic > 10	*P*^2^ < .05
AR to gene	*P*^1^ < 1e−4, kb = 10,000, *r*^2^ < 0.1, maf > 0.01, *F* statistic > 10	*P*^2^ < .05
Immune cells to AR	*P*^1^ < 5e−6, kb = 10,000, *r*^2^ < 0.001, maf > 0.01, *F* statistic > 10	*P*^2^ < .01
Gene to immune cells	*P*^1^ < 5e−6, kb = 10,000, *r*^2^ < 0.1, maf > 0.01, *F* statistic > 10	*P*^2^ < .01

IVW = inverse variance weighted, maf = minor allele frequency, MR = Mendelian randomization, *P*^1^ = The *P*-value of SNPs used as the instrumental variable, *P*^2^ = the *P*-value of MR analysis with IVW, SNPs = single nucleotide polymorphisms.

The data analysis software used in the study was R 4.3.2 (R Foundation for Statistical Computing, Vienna, Austria) and SMR 1.3.1 (The University of Queensland, Brisbane, Australia; https://cnsgenomics.com/software/smr).

## 3. Results

### 3.1. Gene set 1

**Figure 1.**
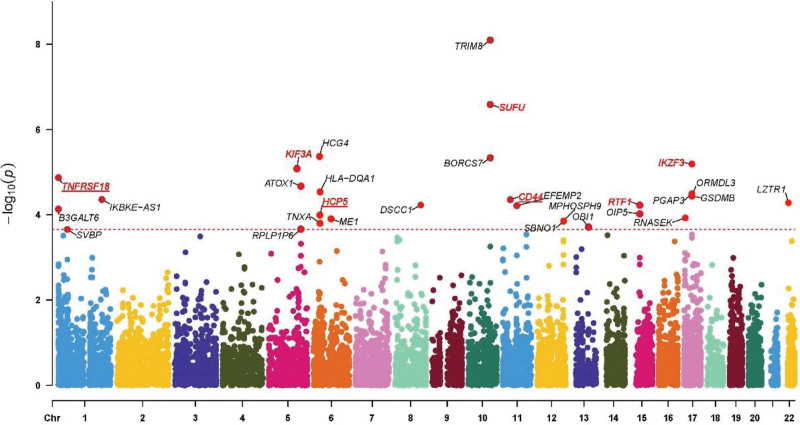
SMR analysis of genetic Manhattan map. The abscissa marks the position of genes on the chromosome, the ordinate represents the corrected *P*-value of the SMR analysis for each gene. All genes shown in the text belong to Gene set 1; genes marked in red belong to Gene set 2; genes highlighted in red and underlined belong to Gene set 3. SMR = summary data based Mendelian randomization.

SMR analysis was performed to identify genes with a causal relationship with AR using the blood cis-eQLTs data from the eqltGen database. A total of 29 genes were identified: TRIM8, SUFU, HCG4, BORCS7, IKZF3, KIF3A, TNFRSF18, ATOX1, HLA-DQA1, PGAP3, GSDMB, ORMDL3, IKBKE-AS1, CD44, LZTR1, DSCC1, RTF1, EFEMP2, B3GALT6, OIP5, HCP5, RNASEK, ME1, MPHOSPH9, SBNO1, TNXA, OBI1, RPLP1P6, and SVBP (genes shown in the text in Fig. 1).

### 3.2. Gene set 2

We obtained a total of 2410 genes from TTD, PharmGkb, GeneCards, DrugBank, and DisGeNet databases, then intersected with the genes in Gene set 1 to obtain 10 genes: SUFU, IKZF3, KIF3A, TNFRSF18, HLA-DQA1, GSDMB, ORMDL3, CD44, RTF1, HCP5 (genes marked in red in Fig. 1).

### 3.3. Gene set 3

**Figure 2.**
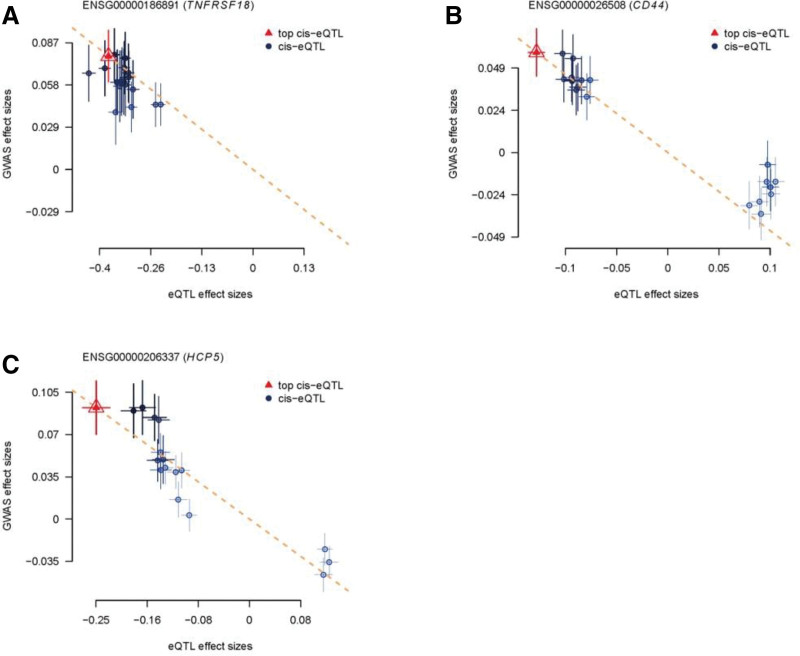
Causal effects of genes on AR based on cis-eQTL in scatterplots. (A) Causal effect of TNFRSF18 on AR; (B) causal effect of CD44 on AR; (C) causal effect of HCP5 on AR. AR = allergic rhinitis, cis-eQTL, cis-expression quantitative trait loci, GWAS = genome-wide association study.

After colocalization analysis of 10 genes in Gene set 2, 3 genes were found to have colocalization relationships with AR, namely TNFRSF18, CD44, and HCP5 (genes highlighted in red and underlined in Figure 1). The posterior probabilities of their H4 hypothesis were 98%, 92%, and 93.7%, respectively. Scatter plots show the relationship between these 3 genes and the risk of AR (Fig. 2), the effect sizes of eQTLs of the 3 genes were all negatively correlated with the effect size of AR, suggesting that with the increase of genes expression, the risk of disease is reduced.

## 4. MR for the mediation of immune cells

### 4.1. Step 1: gene and AR

In Gene set 3, only TNFRSF18 had a significant causal relationship with AR in a 2-sample MR analysis (IVW, OR = 0.846, 95% CI = 0.808–0.886, *P* < .001), there was no pleiotropy because the *P*-value of MR egger intercept was >.05. When adjusting the direction of TNFRSF18 and AR for the reverse MR analysis, only 2 SNPs were found to be eligible for the IVW method, with a *P*-value > .05, as shown in Table S2, Supplemental Digital Content, https://links.lww.com/MD/R707.

### 4.2. Step 2: immune cells to AR

**Figure 3.**
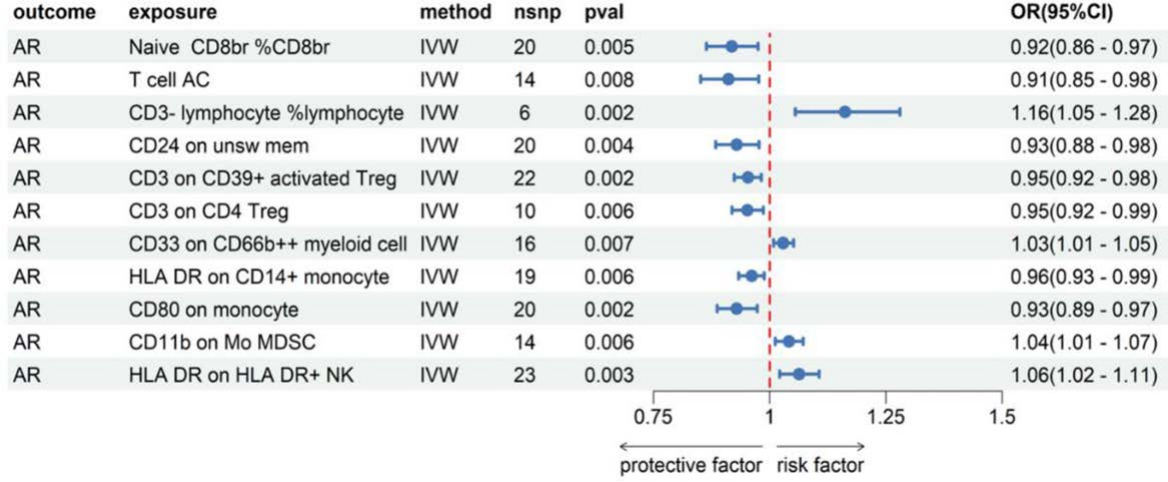
Results of MR analysis of immune cells with a significant causal relationship to AR using IVW method. AR = allergic rhinitis, CI = confidence interval, IVW = inverse-variance weighted, MR = Mendelian randomization, nsnp = number of single nucleotide polymorphisms, OR = odds ratio, *P*-val = value of *P*.

Eleven traits of immune cells that met the criteria were screened: Naive CD8br% CD8br, T cell AC, CD3 lymphocyte% lymphocyte, CD24 on unsw mem, CD3 on CD39+ activated Treg, CD3 on CD4 Treg, CD33 on CD66b++myoid cell, HLA DR on CD14+ monocyte, CD80 on monocyte, CD11b on Mo MDSC, HLA DR on HLA DR+NK. The *P*-values of MR egger intercept were all >.05, which proved that the MR results did not have pleiotropy. The MR results with IVW method of this step are shown in Figure 3.

### 4.3. Step 3: gene to immune cells

We performed TNFRSF18 and 11 traits of immune cells in step 2 with MR analysis, then obtained 3 positive results: CD3 on CD39+ activated Treg (IVW, OR = 1.173, 95% CI = 1.046–1.314, *P* = .006), CD3 on CD4 Treg (IVW, OR = 1.204, 95% CI = 1.074–1.35, *P* = .001), HLA DR on HLA DR+NK (IVW, OR = 1.225, 95% CI = 1.089–1.377, *P* < .001). The pleiotropy of the results was excluded by the MR egger method. However, if HLA DR on HLA DR+NK served as a mediator, the direction of the mediating effect was opposite to that of the total effect, therefore, our subsequent analysis would exclude it.

### 4.4. Step 4: calculation of mediation effect

**Figure 4.**
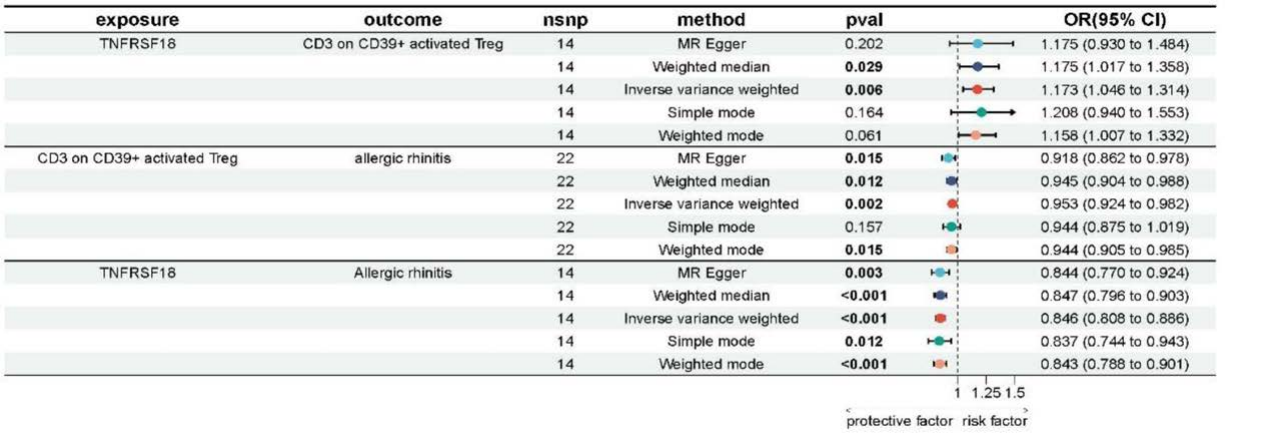
Results of MR analysis for the mediation when CD3 on CD39+ activated Treg acted as the mediator. Bold values indicate statistically significant results (*P* < .05). CI = confidence interval, MR = Mendelian randomization, nsnp: number of single nucleotide polymorphisms, OR = odds ratio, *P*-val = value of *P*.

**Figure 5.**
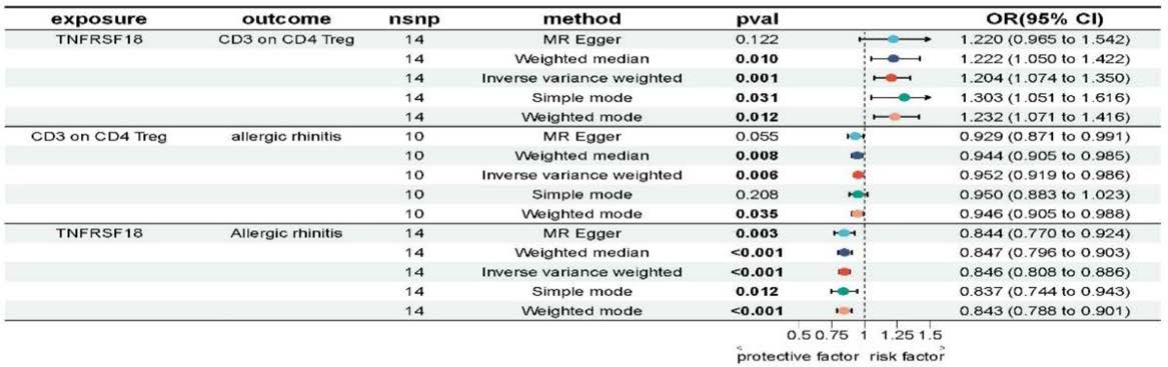
Results of MR analysis for the mediation when CD3 on CD4 Treg activated Treg acted as the mediator. Bold values indicate statistically significant results (*P* < .05). CI = confidence interval, MR = Mendelian randomization, nsnp = number of single nucleotide polymorphisms, OR = odds ratio, *P*-val = value of *P*.

At last, in our MR analysis for the mediation, TNFRSF18 was used as the exposure, CD3 on CD39+ activated Treg and CD3 on CD4 Treg were used as mediators. TNFRSF18 had a positive regulatory effect on the 2 immune cell traits (OR > 1), while the 2 immune cell traits had a negative regulatory effect on AR (OR < 1). The detailed analysis data are shown in Figures 4 and 5. “Leave-one-out” sensitivity analysis indicated that there was no outlier SNP in each step of MR analysis for the mediation (Fig. S1, Supplemental Digital Content, https://links.lww.com/MD/R707).

When CD3 on CD39+ activated Treg served as a mediator, the beta_ab was -0.008, the beta_dir was −0.159, and the beta_p was 0.046; When CD3 on CD4 Treg served as a mediator, the beta_ab was −0.009, the beta_dir was −0.158, and the beta_p was 0.055. Detailed data are presented in Table 2.

**Table 2:** The mediating effect of 2-step MR mediation analysis.

Exposure	Mediator	Outcome	Beta_a	Beta_b	Beta_ab	Beta_c	Beta_dir	Beta_p
TNFRSF18	CD3 on CD39+ activated Treg	AR	0.159	−0.049	−0.008	−0.167	−0.159	0.046
TNFRSF18	CD3 on CD4 Treg	AR	0.186	−0.049	−0.009	−0.167	−0.158	0.055

AR = allergic rhinitis, MR = Mendelian randomization.

## 5. Discussion

To investigate the impact of gene differences on the transcriptome, we selected cis-eQTLs as instrumental variables. In this study, we first integrated the cis-eQTL data in blood using the SMR method to preliminarily identify the genes in blood that impact AR at the genetic level. Then, to increase the reliability of the study, we further searched for the predicted genes related to AR from TTD, PharmGkb, GeneCards, DrugBank, and DisGeNet. Considering both approaches together, we obtained 10 genes related to AR. We then used the colocalization method in a Bayesian framework, which assumes that there are 2 features and at most one causal variation feature. If the hypotheses of association with both traits at a shared causal variant (H4) hold, we believe that there was a colocalization relationship between the 2 traits.^[12]^ After colocalization analysis, TNFRSF18, CD44, and HCP5 genes were found to be associated with AR, suggesting that certain drug components may be able to combine these gene fragment regions to produce therapeutic effects on AR. In addition, we used a 2-sample MR method to extract cis-eQTL data of TNFRSF18 from the ieu open GWAS project to investigate the impact of immune cells on AR. In the 2-step MR for the mediation analysis, in order to maintain consistency in the research method, we extracted 1000 kb SNPs upstream and downstream of the TNFRSF18 gene region from the ieu open GWAS project as instrumental variables.

CD44 is an immune response-related gene, a cytokine-related gene, and also a differential gene between AR and healthy controls.^[13]^ Compared with healthy controls, immune response-related genes are significantly enriched in AR. Among them, the levels of IL1RL1, CD274, and CD44 were significantly higher in AR patients.^[13]^ Meanwhile, Zhang et al^[14]^ also found that CD44 can serve as a differential gene between the AR and control groups, and is still present in the core genes screened by the PPI network and module Molecular Complex Detection (MCODE). However, a discrepancy arises as follows: they pointed out that CD44 is downregulated in the AR group. This divergence may stem from differences in sample types (tissue biopsy vs brush-derived cells) or analytical approaches, highlighting the need for standardized methodologies in AR transcriptomic studies. Our study aligns with the latter finding,^[14]^ where CD44 was identified as a protective factor for AR. However, further studies are needed to validate its role. HCP5 is a long-chain noncoding RNA and was found to be significantly downregulated in peripheral blood mononuclear cells, CD4(+) T cells, Regulatory T cells (Tregs), nasal tissue, and IL-13 treated nasal epithelial cells in AR patients. HCP5 promotes the differentiation and proliferation of Tregs in AR by targeting the miR-16/ATXN2L axis. In addition, the regulation of IL-13 induced nasal epithelial cell dysfunction by lncRNA HCP5 depends on miR-16/ATXN2L in AR inflammatory response.^[15]^ This is consistent with the results of our study, where HCP5 can act as a protective factor to inhibit the development of AR.

TNFRSF18 is an AR-related gene predicted by the GeneCards website, also known as Tumor Necrosis Factor Receiver superfamily member 18 or glucocorticoid induced TNFR-related protein (GITR). It is the only gene in our study that had a significant causal relationship with AR through SMR, colocalization, and 2-sample MR analysis. TNFRSF18 is mainly expressed at high levels in Tregs,^[16]^ and is a key gene that promotes Treg proliferation and mediates immune tolerance.^[17]^ Moreover, TNFRSF18 is expressed not only in T cells, but also in monocytes, macrophages, neutrophils, DC cells, B cells, and NK cells, as well as mast cells, and its expression levels increase after activation, inflammation, or autoimmune processes.^[18]^ In the TNFRSF18 knockout experiment in mice, it was shown that the gene plays a role in the regulation of CD3-driven T cell activation and programmed cell death,^[19]^ CD3-activated T lymphocytes express higher levels of interleukin-2 receptors, and produce a greater amount of interleukin-2.

Research has shown that after 1 year of desensitization treatment, TNFRSF18 levels in the blood of patients with AR decreased, but the difference was not statistically significant.^[20]^ TNFRSF18 is highly expressed in Treg cells. Flow cytometry analysis showed that Foxp3+GITR (TNFRSF18)+Treg cells in rats in the AR model group were significantly lower than those in the normal group and the adjuvant group. Compared with the AR model group, intranasal immunotherapy significantly increased the frequency of Foxp3+GITR (TNFRSF18)+Treg cells.^[21]^ Group 2 innate lymphoid cells (ILC2s) are considered a key control factor for type 2 inflammation and are highly elevated in AR. According to Nagashima et al, anti-TNFRSF18 antibodies boosted the induction of IL-5 and IL-13 in IL-33-mediated human ILC2s and promoted TNFRSF18 expression on ILC2s in response to glucocorticoids. The increased IL-33-mediated activation of IL-5 and IL-13 in human ILC2s implies a connection between the control of AR inflammation and TNFRSF18 expression.^[22]^ Treg/Th17 imbalance is associated with allergic inflammation, including AR, where Treg levels in the peripheral blood of AR patients are significantly reduced.^[23]^ In our study, CD3 on CD39+ activated Treg and CD3 on CD4 Treg showed a protective effect against AR. Based on previous studies, we speculate that TNFRSF18 may be expressed in CD39+ activated Treg and CD3 on CD4 Treg, and may exert immune tolerance by promoting the proliferation of these 2 Treg cells, thereby inhibiting the development of AR.

Although we performed multiple verifications on the gene screening, the results were relatively reliable, however, this study also has some limitations. The research subjects we selected were all from the European population, and further research is needed to determine whether the research results are applicable to other populations. In the reverse MR analysis of TNFRSF18 and AR, even if *P* < 1e−4 was set as the extraction instrumental variable, after screening for strong instrumental variables with *F* statistic > 10, only 2 SNPs met the condition, which resulted in only the IVW method being used for MR analysis and the inability to perform pleiotropy tests.

## 6. Conclusion

In summary, our study found that TNFRSF18, HCP5, and CD44 are genes causally associated with AR and may serve as drug targets to regulate the development of AR through multiple verifications. TNFRSF18 has more sufficient evidence, and it can inhibit the progression of AR by upregulation CD3 on CD39+ activated Treg and CD3 on CD4 Treg immune cells. Future research should further validate these findings through cell-based assays and animal models, and explore their potential for clinical translation – such as developing targeted therapies and diagnostic markers for AR.

## Acknowledgments

We would like to acknowledge the participants and investigators of the FinnGen consortium, eQTLGen consortium, and the study by Orrù’s team for providing eQTLs and GWAS data. We also thank TTD, PharmGkb, GeneCards, DrugBank, and DisGeNet for contributing data. We also thank Dr Zhang Lin for providing statistical consultation for this study. His professional advice significantly improved the accuracy and reliability of our statistical analysis.

## Author contributions

**Conceptualization:** Suhua Chen.

**Data curation:** Jianghua Peng, Xin Yan, Mingzhu Shen.

**Formal analysis:** Xin Yan, Mingzhu Shen.

**Funding acquisition:** Jianghua Peng.

**Investigation:** Jianghua Peng, Xin Yan.

**Methodology:** Xin Yan.

**Project administration:** Suhua Chen.

**Resources:** Mingzhu Shen, Junmei Xuan.

**Software:** Xin Yan.

**Supervision:** Mingzhu Shen, Suhua Chen.

**Validation:** Jianghua Peng, Junmei Xuan, Suhua Chen.

**Visualization:** Junmei Xuan, Suhua Chen.

**Writing – original draft:** Jianghua Peng.

**Writing – review & editing:** Jianghua Peng, Junmei Xuan.

## Supplementary Material


