# Supplementary material for: Identification of allergic rhinitis-related genes and mediating immune cells based on cis-eQTL: A Mendelian randomization study
Source: Medicine (Baltimore). 2026 Apr 17;105(16):e48349. doi: 10.1097/MD.0000000000048349 (PMC13095335; doi:10.1097/MD.0000000000048349)
Supplement: Supplementary file 1 [file medi-105-e48349-s001.pdf]

Supplementary Table 1. Database URL

Database	URL
FinnGen（R10）	https://www.finngen.fi/en/access_results
eQTLGen	https://www.eqtlgen.org/
ieu open GWAS	https://gwas.mrcieu.ac.uk/
TTD	https://db.idrblab.net/ttd/
PharmGkb	https://www.pharmgkb.org/
GeneCards	https://www.genecards.org/
DisGeNet	https://www.disgenet.org/
DrugBank	https://go.drugbank.com/

URL: Uniform Resource Locator.

Supplementary Table 2. MR Results of AR to TNFRSF18

Outcome	Exposure	Method	Nsnp	*P*-value	OR	95%CI
TNFRSF18	AR	IVW	2	0.522	0.911	0.685-1.212

MR: Mendelian Randomization; AR: allergic rhinitis; Nsnp: number of single nucleotide polymorphisms; OR: odds ratio; CI: confidence intervals.

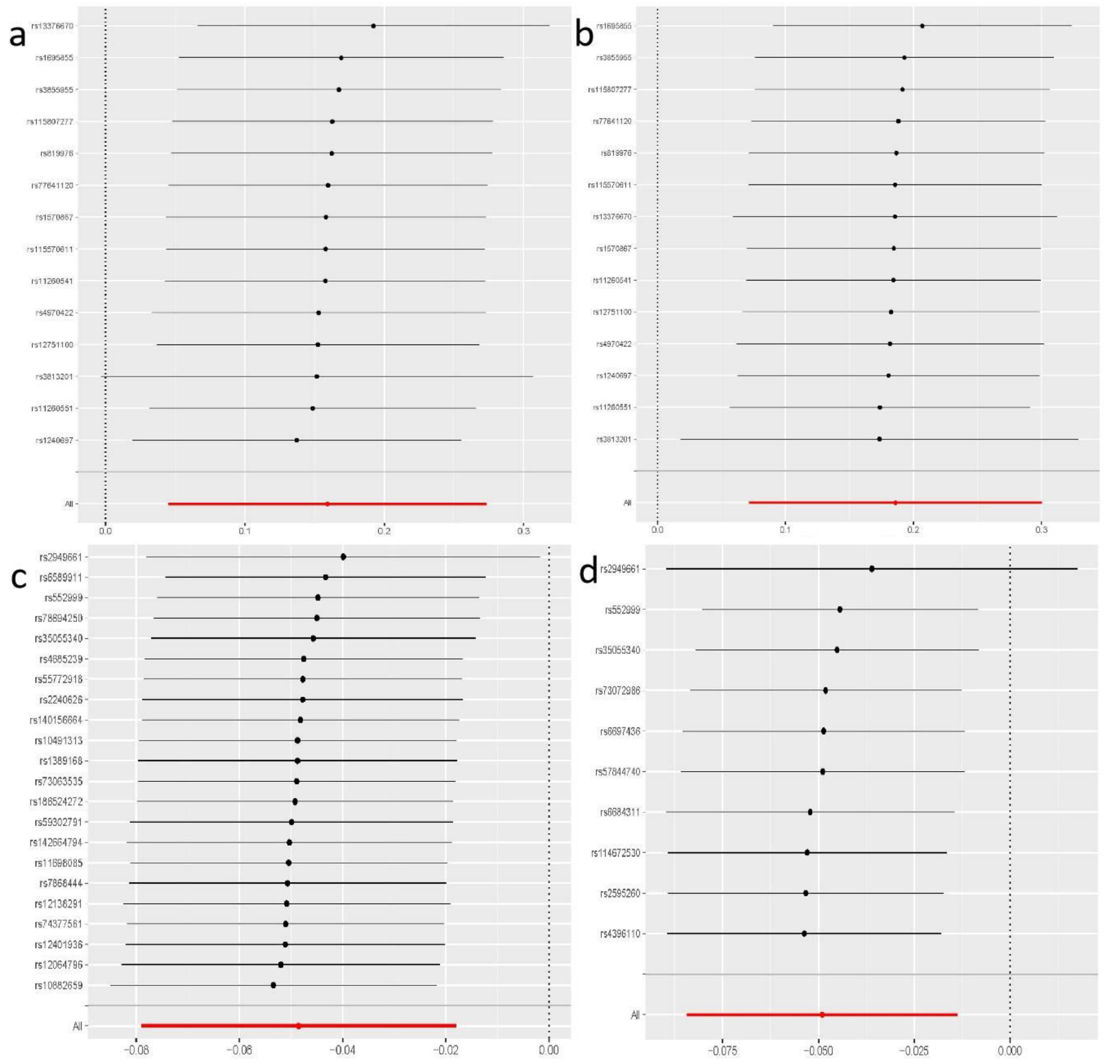

Supplementary Figure 1. MR leave-one-out sensitivity analysis. Panel a is for “TNFRSF18” on “CD3 on CD39+ activated CD4 Treg”, b is for “TNFRSF18” on “CD3 on CD4 Treg”, c is for “CD3 on CD39+ activated CD4 Treg” on “Allergic rhinitis”, d is for “CD3 on CD4 Treg” on “Allergic rhinitis”. MR: Mendelian randomization.
